# Evaluation of a remote-controlled laparoscopic camera holder for basic laparoscopic skills acquisition: a randomized controlled trial

**DOI:** 10.1007/s00464-020-07899-5

**Published:** 2020-08-26

**Authors:** Mohammad S. A. Amin, Abdullatif Aydin, Nurhan Abbud, Ben Van Cleynenbreugel, Domenico Veneziano, Bhaskar Somani, Ali Serdar Gözen, Juan Palou Redorta, M. Shamim Khan, Prokar Dasgupta, Jonathan Makanjuoala, Kamran Ahmed

**Affiliations:** 1 MRC Centre for Transplantation, King’s College London, Guy’s Hospital, King’s Health Partners, London, SE1 9RT UK; 2 Department of Urology, University Leuven, Leuven, Belgium; 3 Department of Urology and Renal Transplantation, Bianchi-Melacrino-Morelli Hospital, Reggio Calabria, Italy; 4 Department of Urology, Southampton University Hospital NHS Foundation Trust, Southampton, UK; 5 Department of Urology, SLK-Kliniken, University of Heidelberg, Heilbronn, Germany; 6 Department of Urology, Fundació Puigvert, Universitat Autònoma de Barcelona, Barcelona, Spain; 7 Urology Centre, Guy’s and St. Thomas’ NHS Foundation Trust, London, UK; 8 Department of Urology, King’s College Hospital NHS Foundation Trust, London, UK; 9 European School of Urology (ESU) Training and Research Group, Barcelona, Spain

**Keywords:** Robotic camera holder, Laparoscopy, E-BLUS, Surgical skills, Human error

## Abstract

**Background:**

Unsteady camera movement and poor visualization contribute to a difficult learning curve for laparoscopic surgery. Remote-controlled camera holders (RCHs) aim to mitigate these factors and may be used to overcome barriers to learning. Our aim was to evaluate performance benefits to laparoscopic skill acquisition in novices using a RCH.

**Methods:**

Novices were randomized into groups using a human camera assistant (HCA) or the FreeHand v1.0 RCH and trained in the (E-BLUS) curriculum. After completing training, a surgical workload questionnaire (SURG-TLX) was issued to participants.

**Results:**

Forty volunteers naïve in laparoscopic skill were randomized into control and intervention groups (*n* = 20) with intention-to-treat analysis. Each participant received up to 10 training sessions using the E-BLUS curriculum. Competency was reached in the peg transfer task in 5.5 and 7.6 sessions for the ACH and HCA groups, respectively (*P* = 0.015), and 3.6 and 6.8 sessions for the laparoscopic suturing task (*P* = 0.0004). No significance differences were achieved in the circle cutting (*P* = 0.18) or needle guidance tasks (*P* = 0.32). The RCH group experienced significantly lower workload (*P* = 0.014) due to lower levels of distraction (*P* = 0.047).

**Conclusions:**

Remote-controlled camera holders have demonstrated the potential to significantly benefit intra-operative performance and surgical experience where camera movement is minimal. Future high-quality studies are needed to evaluate RCHs in clinical practice.

**Trial registration:**

ISRCTN 83733979

Many technical limitations of laparoscopic surgery are overcome with robot-assisted surgery, but due to socioeconomic factors laparoscopy remains the favoured modality for many trusts [1]. However, despite laparoscopy’s widespread application most research has focused on improving robot-assisted surgery. As such, some of the disadvantages of laparoscopic surgery e.g. operation duration, complication, and mortality still exist and may be improved upon by addressing the challenges of the learning curve (LC) [2–7].

Laparoscopy’s difficult LC is attributed to the increased workload when compared to open surgery as maintaining challenging physical positions increases stress, physical demand, and reduces performance [8–10]. Furthermore, errors made by human camera assistants (HCAs) due to inexperience, miscommunication, tremor, involuntary rotation of the camera’s axis, erroneous movements, and fatigue contribute to an unsteady camera image which can also reduce surgical performance [5, 9, 11–17].

Improvements to camera assistance may facilitate a shorter learning curve so trainee surgeons can gain competency quicker to treat patients. In the 1990s prior to the advent of telesurgical robots, remotely controlled robotic camera holders were introduced to produce stable images and fewer inadvertent movements, [5, 10, 11, 15, 16, 18, 19]. As remote-controlled camera holders (RCHs) eliminate tremor, camera rotation, and muscular fatigue they may be advantageous when compared to conventional HCAs. However, few high-quality studies have taken place evaluating their performance benefits.

This study aims to evaluate the benefits of a RCH and by mapping it's LC, identify potential challenges experienced [2, 3].

## Materials and methods

### Trial design

**Fig. 1:**
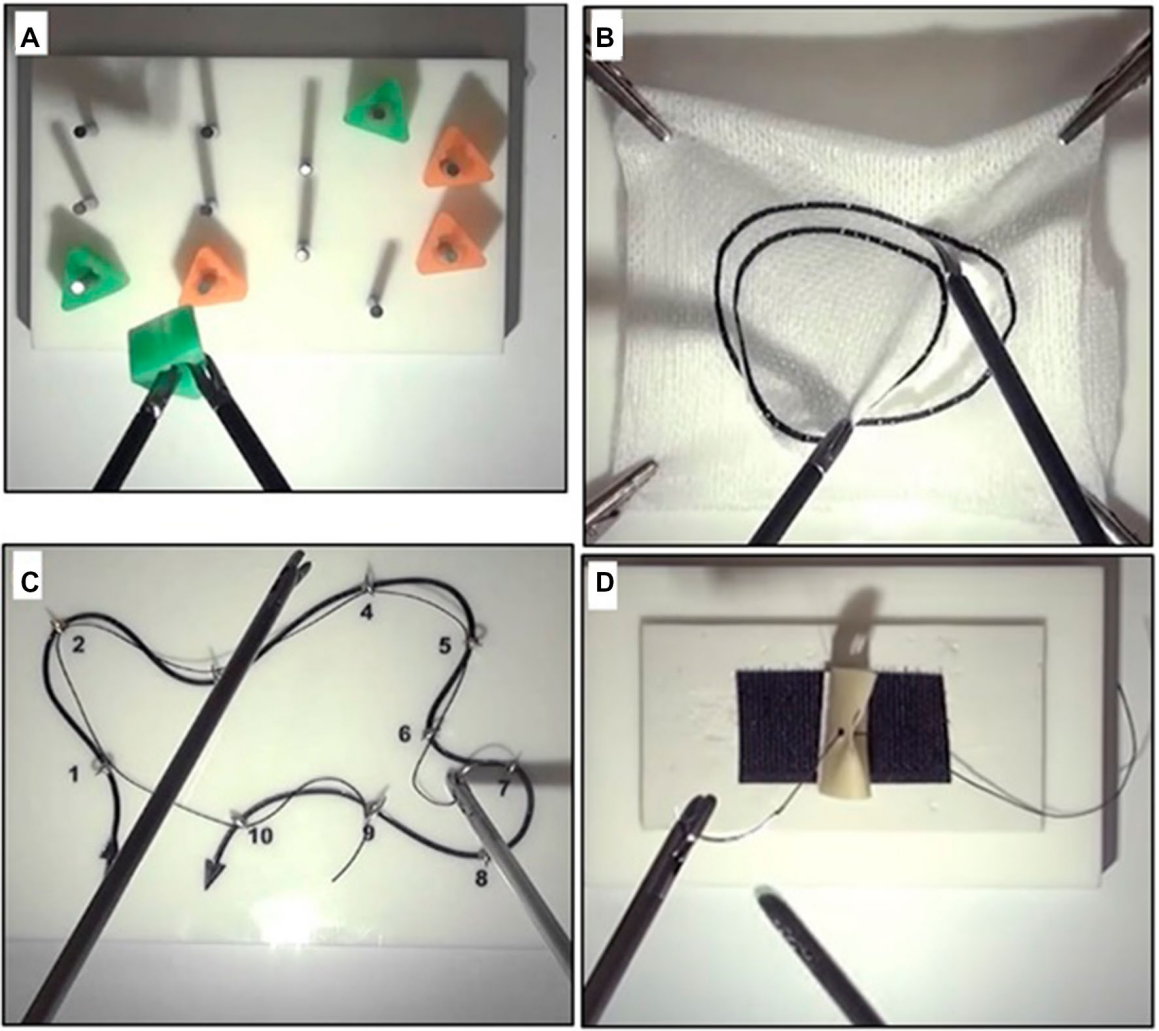
Individual tasks of the European Training In Basic Laparoscopic Urological Skills (E-BLUS) curriculum. **a** Peg transfer. **b** circle cutting. **c** needle Guidance. **d** laparoscopic suturing

This study was a prospective randomized controlled trial designed according to the Consolidated Standards of Reporting Trials (CONSORT) 2010 guidelines [20]. Novice participants (medical students naïve in laparoscopic theory, laparoscopic practical skill, and camera holding) were enrolled onto a basic laparoscopic skills (BLS) training course where the four tasks of the validated European training in basic laparoscopic skills (E-BLUS) curriculum were practiced (Fig. 1).

All participants attended a mandatory induction course for theoretical teaching and demonstration of technique required to pass E-BLUS tasks. Skill demonstration was performed by MSAA and NA who were trained in laparoscopic technique and E-BLUS competent. The opportunity to ask questions on E-BLUS and camera holding technique was given [2, 21]. Supplementary e-learning resources on LS theory and technique for performing E-BLUS tasks were distributed [3].

Skill sessions were modelled on a training curriculum for simulated laparoscopic cholecystectomy in novices [2]. Tasks were performed once per skills session with a limit of two per day with no opportunity for warmup [22]. One-hour breaks were implemented between sessions with a maximum of ten being offered [2, 4, 23, 24]. Terminal participant-specific feedback was consistently provided by the skill demonstrators to remind participants the appropriate technique and to increase economy of movement [4, 25].

Trial participants were also used as HCAs in the control group to replicate the conventional practice of having a human hold the laparoscope. Initially naïve in camera holding, all novices received the same level of dedicated training during a mandatory induction course to standardize their impact to participants’ performance during skills sessions. As best to our knowledge, no data exist on when camera competency is achieved so this was practiced until HCAs reached a proficient standard determined by their ability to efficiently visualize all possible areas of the box trainer with appropriate technique.

### Intervention and materials

E-BLUS tasks were performed using training platforms (Intech, Calabria, Italy), folded gauze with printed circles, and penrose drains (Limbs & Things, United Kingdom). 4–0 polypropylene sutures were used for needle guidance and suturing.

**Fig. 2:**
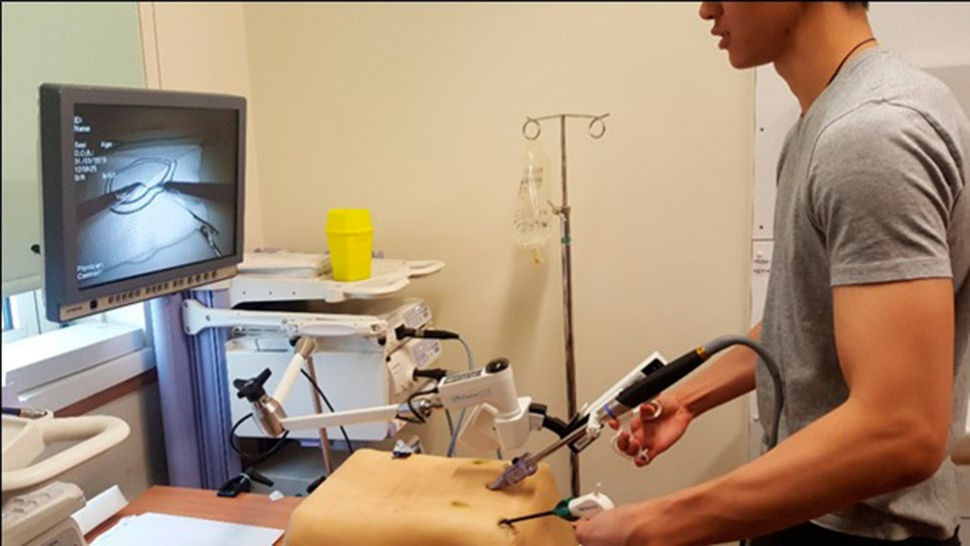
A participant using the FreeHand automated camera holder

The experimental group used the FreeHand® v1.0 RCH (FreeHand Ltd, Guildford, United Kingdom) which is favoured by surgeons and free of safety concerns (Fig. 2) [5, 10, 18]. It is a robotic arm specifically designed for holding laparoscopes and cable of movement in 3 planes. It was controlled with a joypad by a human operator and was moved only under direct instructions of the participant.

The RCH requires minimal practice to gain competency and so operators practiced until they felt comfortable [16, 18]. A supplementary guide on how to use the RCH was also issued.

### Outcomes

#### Primary outcome

The primary outcome was the time taken to reach competency in E-BLUS tasks, defined as maintaining the pass score for at least two consecutive sessions [2]. To map the learning curve an expert developed, performance improvement (Pi) score described by Veneziano et al. was used [25]. The Pi score measured skill by comparing task duration and errors made, to previous performances in relation to the required pass mark [25]. It was not feasible to use blinded outcome assessors as the smooth movement and absence of tremor from the RCH would be easily discernible. Thus, the outcomes were assessed by the skill demonstrators.

#### Secondary outcome

The secondary outcome was the surgical workload experienced by participants measured by the validated SURG-TLX questionnaire distributed after the final skills session [8].

### Sample size and randomization

As no data on E-BLUS learning curves have been previously published, we estimated that benefits of the RCH would decrease the required number of skills sessions to gain competency from six to four. These variables were used in a sample size calculation for a test comparing two independent means at an alpha level of 0.05 and power of 80%. Using StataIC 15 (StataCorp, Texas, USA), the calculation yielded a size of six participants in each group. The number of participants was raised to 20 in accordance with methods of a previous learning curve study to accommodate for dropout and increase trial accuracy [2]. Limitations to study resources prevented further participants from being recruited into the BLS course and this study.

60 eligible participants were randomly selected from the recruitment form and block randomized into three groups at a ratio of 1:1:1: via randomizer.org. Groups were then randomly assigned control or intervention status. Only the control group and one intervention group are the focus of this study. Enrolment of participants, assignment of interventions, and randomization of participants were performed by MSAA. Participants were blinded to randomization but not the intervention.

### Statistical methods

Unpaired *t* tests were used to compare the scores of both the groups to identify statistical significance between primary and secondary outcomes. To exclude unreliable reporting of results from the SURG-TLX questionnaire, outlier scores were removed prior to unpaired *t* tests [26, 27].

### Results

#### Participant flow and recruitment

**Fig. 3:**
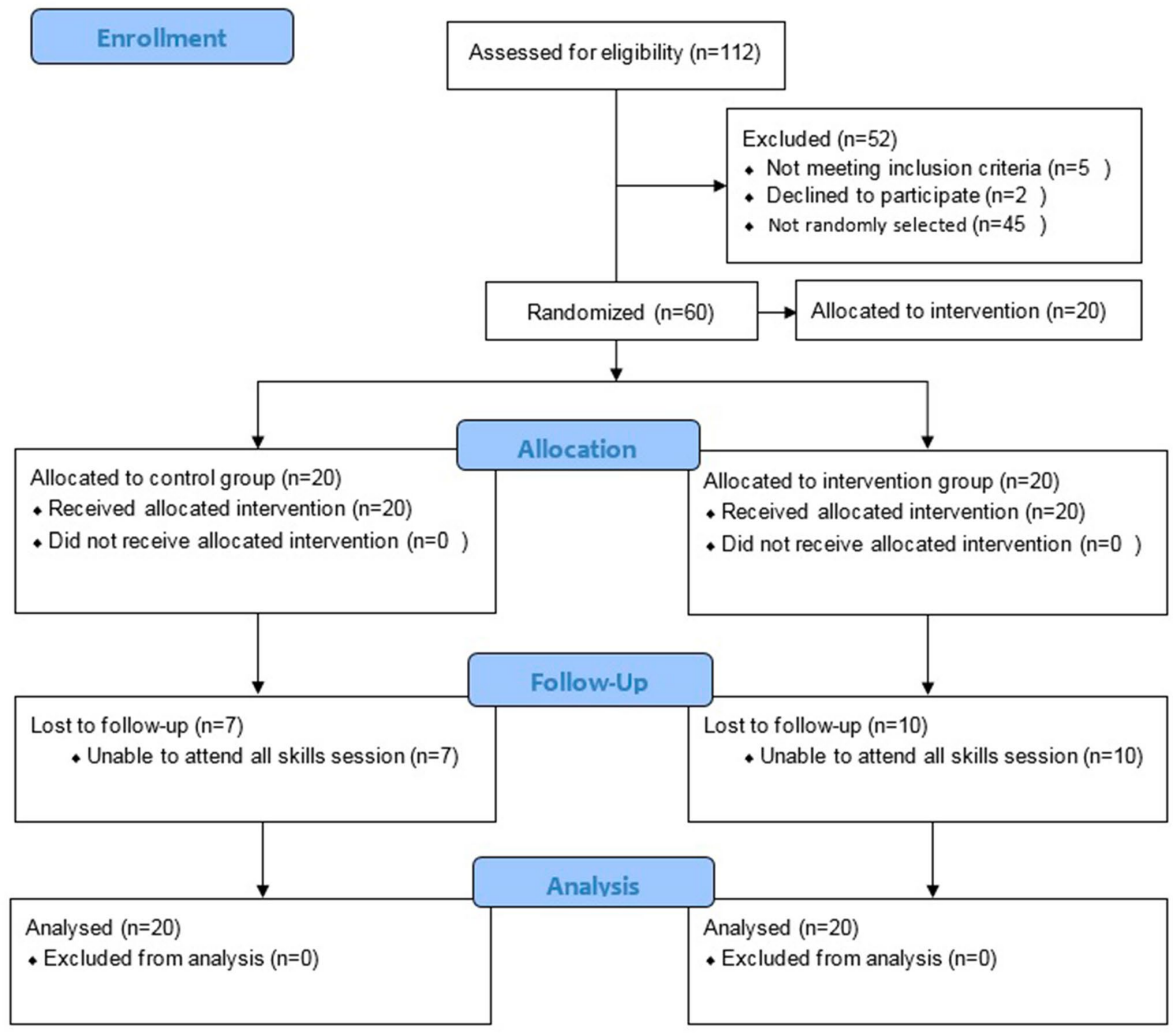
Consort 2010 flow diagram of participants for the primary outcome

The skills course received 112 applications of which 60 were evenly randomized into one control and two intervention groups. The participants who were not randomized into the RCH or HCA groups were allocated a different intervention which used the Microsoft HoloLens and do not form part of this trial. All participants in both groups received their allocated intervention with 13 participants from the control group and 10 from the intervention group completing all 10 sessions. Data were gathered for intention-to-treat analysis for which data from all 40 participants were analysed (Fig. 3). The trial ended when participants completed all 10 skill sessions or were lost to follow-up.

### Baseline data

Demographic data were collected from participants via the recruitment form. This consisted of age, gender, year of medical study, previous laparoscopic experience, and interest in laparoscopic skill. Differences in demographics between groups were not statistically significant (Table 1).

**Table 1:** Demographic data of trial participants

Characteristic	Human camera assistant group (HCA) *n* = 20	Remote-controlled camera holder group (RCH) *n* = 20
Mean age (range)	21.8 (18–28)	21.2 (18–26)
Gender	Male: 6	Male: 8
	Female: 14	Female: 12
Year of medical study	1st: 5	1st: 5
	2nd: 3	2nd: 5
	3rd: 9	3rd: 7
	4th: 2	4th: 2
	5th: 0	5th: 0
	6th: 1	6th: 1
Laparoscopic interest	Yes: 20	Yes: 20
	No: 0	No: 0
	Undecided: 0	Undecided: 0
Previous laparoscopic experience (theoretical, practical, laparoscope holding)	Yes: 0	Yes: 0
	No: 20	No: 20

### Primary outcome: basic laparoscopic skill

**Fig. 4:**
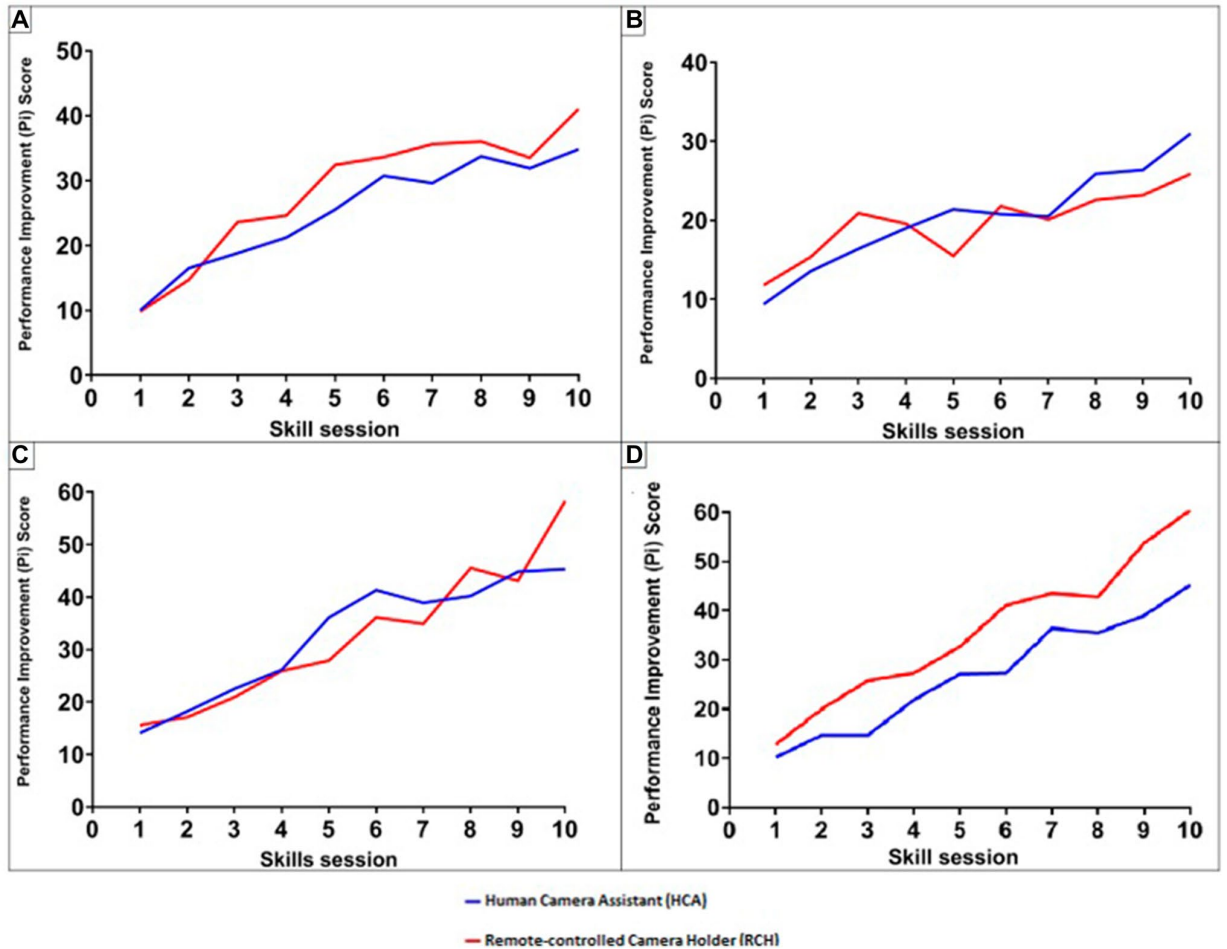
Learning curves of the European training in basic laparoscopic urological skills (E-BLUS) tasks. **a** Peg transfer. **b** circle cutting. **c** Needle guidance. **d** Laparoscopic suturing

The initial Pi scores were similar for both groups in all tasks (Fig. 4). In the peg transfer (PT) task, the RCH surpassed the HCA at the 3rd mean session and gained competency significantly faster at 5.5 and 7.6 mean sessions, respectively (*P* = 0.015) (Table 2). In the circle cutting (CC) task, both groups experienced widespread plateaus. By the 5th mean session, the HCA held a decisive lead over the RCH. Only four participants of the HCA group gained competency compared to the two of the RCH group. This difference was not statistically significant (*P* = 0.18). Both groups progressed rapidly in the needle guidance (NG) task. After the 4th mean session, the HCA surpassed the ACH, however, by the 8th mean session, the RCH had an overall score of 58.3 to the HCA’s 45.3. There was no significant difference in the RCH gaining competency by 4.7 mean sessions compared to a mean of 5 sessions of the HCA (*P* = 0.32). In the laparoscopic suturing (LS) task, the RCH performed consistently better than the HCA. The HCA group experienced three plateaus, whereas the RCH group only had one. The RCH group was significantly faster in reaching competency at a mean of 3.6 sessions whilst the HCA group needed a mean of 6.8 sessions (*P* = 0.00038).

**Table 2:** Average number of sessions required to reach competency in individual E-BLUS tasks for the Remote-controlled camera holder (RCH) and human camera assistant (HCA) groups

Task	Sessions taken to reach competency using RCH (range)	Sessions taken to reach competency using HCA (range)	*P* value
Peg transfer	5.5 (2–9)	7.6 (4–10)	*P* = 0.015
Circle cutting	10 (10–10)	9.25 (8–10)	*P* = 0.18
Needle guidance	4.7 (2–9)	5 (3–9)	*P* = 0.32
Laparoscopic suturing	3.6 (1–7)	6.8 (4–10)	*P* = 0.00038

### Secondary outcome: surgical workload

Ten participants from the control group and 13 participants from the intervention completed the SURG-TLX questionnaire. One outlier from the intervention group was removed prior to analysis. Analysis of the remaining data was then performed (Table 3). Total workload score for the RCH group was 170.7, whereas the control group scored 195.8 (*P* = 0.014). In the distraction domain, the RCH group scored 10.8 whilst the HCA group scored 29 achieving borderline significance (*P* = 0.047).

**Table 3:** Average scores of the SURG-TLX questionnaire broken down by domain for the human camera assistant (HCA) and Remote-controlled camera holder (RCH) groups

Domain	HCA	RCH	*P* value
Mental demand	26.60	26.50	*P* = 0.5
Physical demand	46.60	33.67	*P* = 0.093
Temporal demand	35.20	42.17	*P* = 0.21
Task complexity	34.00	32.33	*P* = 0.44
Situational stress	24.40	25.17	*P* = 0.46
Distractions	29.00	10.83	*P* = 0.047
Total workload score	195.80	170.67	*P* = 0.014

### Harms

Some participants experienced temporary anxiety and stress when committing errors or finding it technically difficult to complete a task. Some participants also experienced tiredness and muscular fatigue as they were not accustomed to laparoscopy.

## Discussion

The trial successfully produced a learning curve of basic laparoscopic skills and demonstrated the skills course was effective for all participants to reach competency in the PT, NG, and LS tasks. The largest changes in Pi scores were seen by the first three sessions as more than 50% of the final Pi value had been achieved. Studies suggest that the large initial increases to Pi score is due to refinements in accuracy which reduces errors shortly followed by improvements to time efficiency [21, 25, 28, 29].

The advantages provided by the RCH were evident from the marginal increase in initial task scores when compared to the HCA group. This was further emphasized by the differences in time taken to competency which significantly favoured the RCH group for PT and LS tasks, whereas CC and NG had no significant differences. In the CC task, the HCA had a higher Pi score than the RCH. Yet, most participants did not gain competency and it took at least eight sessions for those who did make it the most difficult E-BLUS task. This difficulty could be attributed to the circle folding back on itself requiring constant reorientation of the scissors. It was expected that tasks would have different LCs as they each required a different combination of skills which both cohorts would have different advantages in e.g. a fixed camera position would have optimized speed in the PT task so it is unsurprising for the RCH group to have performed significantly better here. Performance during PT also benefited by having a view of the entire operative field which similarly improved performance in LS as participants were more capable of simultaneously tying both ends of the suture. Maintaining the same advantageous perspective would have been physically difficult for a HCA further confirming that improper camera control is a limiting factor in laparoscopic performance.

Improvements in task performance may have been contributed by decreased surgical workload as the RCH group experienced lower levels of distraction. This may be due to team members in the HCH group physically obstructing each other when repositioning to complete tasks, a problem not encountered with the RCH allowing for constant focus [10]. This finding was in contrast to a study by Wijsman et al. using a different RCH where the workload in a clinical environment was not affected, a potential reason for this was the use of the NASA-TLX and not the modified SURG-TLX as used in this study [10].

In the NG and CC tasks, the camera angle was continuously changed as different regions required visualization. As humans are much faster in moving the camera this trial shows that the RCH is slightly disadvantaged in situations where constant movement is necessary [16]. To minimize time loss from repositioning the optimum perspective should be found to encourage economy of movement. This minimizes the number of camera movements required and allows for continuous cutting to maximize time efficiency. Furthermore, participants encountered barriers to performance in the NG task as the needle frequently bent contributing to task difficulty. Emphasis should therefore be made to teach appropriate needle handling technique.

As no definitive plateaus were reached within the ten skill sessions further studies to identify the point of task mastery may prove to be beneficial in preparing for more advanced tasks [30]. However, the temporary plateaus and dips in the LC were likely due to participants deviating from the demonstrated methods as they may have attempted to take shortcuts, felt rushed, or experimented with techniques. This was rectified through terminal feedback where reminders of the most efficient techniques were restated. To pre-empt these plateaus there may be benefit in scheduling demonstrations after the initial phase of the LC to consolidate proper technique and prevent this deviation. As barriers to skill acquisition were identified the implantation of technology such as the RCH may be used to overcome these challenges and significantly reduce the learning curve, maximising the cost efficiency and performance of the workforce.

This trial observed that the RCH reduces learning curves in simulation of basic tasks. As BLS training programmes have transferability to clinical practice it is plausible that the RCH may be able to improve the performance of trainees by shortening the intra-operative LC. Benefit may also be extended to experienced surgeons as changes to surgical workload are more pronounced in clinical environments due to increased decision making, anatomical recall, and procedure complexity [4, 24, 30]. The ensuing benefits would be cost efficiency to surgical training as the surgeon can focus on teaching rather than camera handling, additionally clinical practice would improve as the assistant would be free to attend to patients in wards or clinics [2, 5, 9–11, 18, 19].

The trial’s primary strength is the robust skills programme designed to optimize learning, performance, and retention of skill. This was achieved by reviewing high-quality studies of educational techniques e.g. preventing fatigue and methods for delivering feedback. Furthermore, randomizing a large cohort of participants and measuring outcomes over many training sessions served to increase accuracy. Additionally as both cohorts had insignificant differences in their ages their LS ability would have been unchanged [24].

Participants were not screened for previous open suturing experience as there is little transfer into laparoscopic skill, however, previous suturing experience may have been advantageous in understanding LS technique. This risk was minimal due to cohorts being large, randomized, and containing junior medical students where suturing experience is uncommon. Additionally, as two demonstrators were used, learning for individual students may have been different. However, this too was mitigated as tutors agreed on technique prior to teaching. Furthermore, as the demonstrators also assessed the outcomes of the study and were not blinded to the intervention there is a potential risk of bias. This bias was reduced by assessors strictly adhering to the official E-BLUS guidance on when a task begins, is completed, and what constitutes an error.

The use of trial participants as both laparoscopic novices and human camera assistants may have introduced risk of bias to the study as partaking in one task may have enhanced skill in the other. This may have confounded results as camera holding performance may have increased by partaking in the skills course and the participants own laparoscopic skills may have increased from holding the camera. This is extremely unlikely given that camera holding and laparoscopy utilize different skills. To our knowledge, no study identifies a crossover in performance between camera holding and laparoscopic. Any potential bias was decisively addressed as all HCAs were at a proficient level of skill prior to the collection results. Potential gains in skill from laparoscopic exposure would have had limited impact to camera holding ability. Furthermore, the control group creates the conventional environment where an inexperienced assistant often holds the laparoscope [13].

Another limitation was the workload experienced by the assistant was not measured. It is likely a greater difference would have been observed as the primary benefit of the RCH is to improve upon ergonomics of camera holding. An improvement to the trial would have been to also assess performance using an experienced camera assistant [16].

## Conclusion

This study identifies that a RCH significantly benefits novices in BLS particularly when camera movement is minimal. As these results were from a low-fidelity environment future randomized controlled trials should consider performance of intermediates and experts in clinical settings where prolonged operations may emphasize the benefits of RCHs [5] [16, 24]. Additional workload scores and intra-operative ergonomic studies may produce more reliable results of the FreeHand’s benefit as different stressors are prevalent during surgery [28].
